# Biomimetic Cell-Derived Nanoparticles: Emerging Platforms for Cancer Immunotherapy

**DOI:** 10.3390/pharmaceutics15071821

**Published:** 2023-06-26

**Authors:** Tingting Hu, Yuezhou Huang, Jing Liu, Chao Shen, Fengbo Wu, Zhiyao He

**Affiliations:** 1 Department of Pharmacy, Cancer Center and State Key Laboratory of Biotherapy, West China Hospital, Sichuan University, Chengdu 610041, China; tingtinghu2017@scu.edu.cn (T.H.); huangyuezhou@wchscu.cn (Y.H.); jingliu0103@scu.edu.cn (J.L.); shenchao@scu.edu.cn (C.S.); 2 Key Laboratory of Drug-Targeting and Drug Delivery System of the Education Ministry, West China School of Pharmacy, Sichuan University, Chengdu 610041, China

**Keywords:** biomimetic nanoparticles, cell-membrane cloaking, cancer immunotherapy, drug delivery system, nanotechnology

## Abstract

Cancer immunotherapy can significantly prevent tumor growth and metastasis by activating the autoimmune system without destroying normal cells. Although cancer immunotherapy has made some achievements in clinical cancer treatment, it is still restricted by systemic immunotoxicity, immune cell dysfunction, cancer heterogeneity, and the immunosuppressive tumor microenvironment (ITME). Biomimetic cell-derived nanoparticles are attracting considerable interest due to their better biocompatibility and lower immunogenicity. Moreover, biomimetic cell-derived nanoparticles can achieve different preferred biological effects due to their inherent abundant source cell-relevant functions. This review summarizes the latest developments in biomimetic cell-derived nanoparticles for cancer immunotherapy, discusses the applications of each biomimetic system in cancer immunotherapy, and analyzes the challenges for clinical transformation.

## 1. Introduction

Cancer immunotherapy, including immune checkpoint inhibitors (ICIs), adoptive cellular immunotherapy (ACT), cancer vaccines, and cytokine immunotherapy, is an effective treatment method that recognizes and kills cancer cells by activating the autoimmune system [1]. Although cancer immunotherapy has made some achievements in clinical cancer treatment, it is still restricted by systemic immunotoxicity, immune cell dysfunction, cancer heterogeneity, and the ITME [2,3]. In addition, how to accurately deliver therapeutic agents to tumors for enhanced immune responses and reduced systemic immunotoxicity is one of the urgent issues to be solved.

In the past decades, nanoparticles have gradually emerged as promising drug delivery carriers for chemotherapy, photothermal therapy (PTT), gene therapy, and immunotherapy due to their good design flexibility, reduction of side effects, and improvement of in vivo efficacy [4,5,6,7]. However, owing to the complexity of the blood environment in vivo, nanoparticles with high immunogenicity can be easily identified and removed by the mononuclear phagocytic system [8,9]. Engineered nanoparticles show promise for improving the biodistribution and metabolism of nanoparticles. For instance, coating nanoparticles with PEG can prolong their circulation half-life in the blood [10,11]. Nevertheless, long-term repeated PEGylated nanoparticles treatment can, in turn, accelerate their clearance and trigger more intense immunogenic stimulation [12,13]. Therefore, more effective solutions are needed. To address these issues, the application of biomimetic cell-derived nanoparticles is a promising strategy [14,15,16]. 

**Figure 1:**
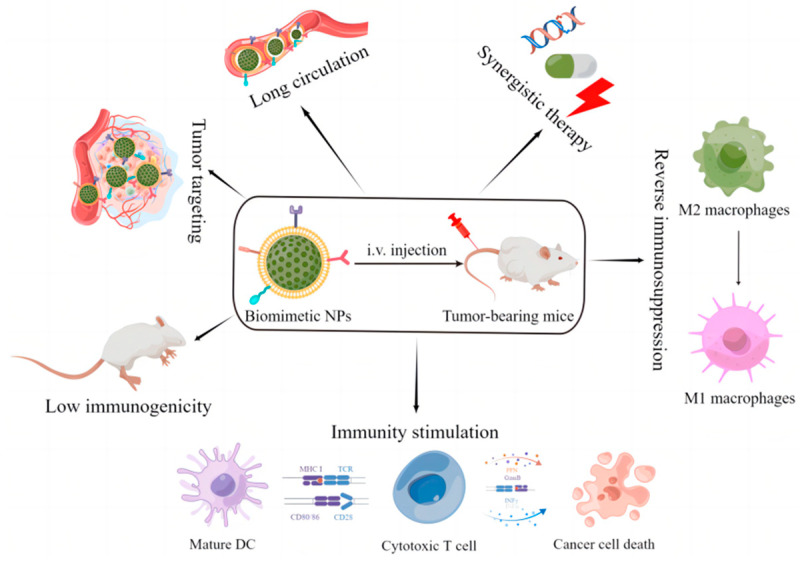
The unique advantages of biomimetic cell-derived nanoparticles. By Figdraw.

In the field of cancer diagnosis and therapy, nanoparticles coated with cell membranes combine the biological functions of cell membranes with the drug-loading capability of engineered core nanoparticles, thereby providing a series of unique advantages (Figure 1), including improved biological interface properties, lower immunogenicity, better biocompatibility, longer circulation, more efficient drug delivery, and elevated active-targeting [17,18,19,20]. Therefore, biomimetic cell-derived nanoparticles have been applied to the precision delivery of theranostic agents or to directly improve the therapeutic efficacy of cancer immunotherapy. 

In this review, we underline the unique advantages of biomimetic cell-derived nanoparticles, summarize the latest developments in biomimetic cell-derived nanoparticles for cancer immunotherapy, and analyze the challenges of clinical translation of biomimetic cell-derived nanoparticles.

## 2. Biomimetic Cell-Derived Nanoparticles

Biomimetic cell-derived nanoparticles are synthetic nanoparticles camouflaged with natural or engineered cell membrane materials to trick the immune system and enhance tumor targeting [21,22]. The structure of biomimetic cell-derived nanoparticles is a core–shell structure, where the core is the nanoparticles delivering the therapeutic agents to the target site and the shell is the membrane materials extracted from different cells. These resulting biomimetic nanoparticles combine the physical and chemical properties of nanoparticles with the intrinsic properties of natural cell membranes, possessing the capacity to evade the immune system, prolong blood circulation, and actively target diagnostic and therapeutic agents to the targeted sites [17,18,19,20]. In 2011, Hu et al. constructed erythrocyte membrane-camouflaged nanoparticles by co-extruding PLGA polymeric nanoparticles and erythrocyte membrane-derived vesicles [23]. This is the first report of nanoparticles derived from cell membranes. Since then, researchers have designed various biomimetic cell-derived nanoparticles to their meet desired functions, and flexibly combined different types of nanoparticles with different sources of biomimetic membrane materials [24,25,26,27], such as cancer cell membranes, white blood cell or leukocyte membranes, stem cell membranes, platelet membranes, and bacteria membranes. The biomimetic cell-derived nanoparticles have been widely recognized for a variety of applications in drug delivery, disease diagnosis, immune modulation, and disease treatment [28,29,30].

### 2.1. Unique Function of Biomimetic Cell-Derived Nanoparticles

Biomimetic cell-derived nanoparticles inherit abundant important biological functions related to their source cells, including “self” labeling, biological targeting, cross-talk with the immune system, and region-specific homing. Most of these important functions are attributed to specific proteins or molecules on the surface of cell membranes.

#### 2.1.1. Erythrocyte Membranes

Erythrocytes can prolong the systemic circulation time of wrapped nanoparticles due to some special membrane proteins [31], and the most important biomarker is CD47. Briefly, signal-regulatory protein alpha (SIRPα) on the surface of phagocytes interacts with CD47 expressed by erythrocytes, which disguises the immune cells as self and prevents immune phagocytes from phagocytizing erythrocytes [32]. In addition, CD59, C8 binding protein (C8bp), and complement receptor 1 (CR1) on the surface of erythrocytes have a role in defending against complement system attack [33]. Therefore, nanoparticles coated with erythrocyte membranes have a prolonged circulation half-life and are less immunogenic. Nevertheless, erythrocyte membranes have no tumor-targeting properties. To remedy this deficiency, researchers further utilize tumor-targeting ligands/peptides to modify erythrocyte membranes or construct erythrocyte membrane-based hybrid membranes to realize tumor targeting [34,35,36,37].

#### 2.1.2. Immune Cell Membranes

Tumors are chronic inflammatory tissues that attract and recruit immune cells by secreting a variety of chemokines and cytokines [38,39]. Therefore, the adhesive property of immune cells can be exploited to actively target drugs for cancer treatment [40].

Dendritic cells (DCs) are professional antigen presenting cells (APCs). Thus, mature DC membrane-wrapped nanoparticles can thoroughly inherit the antigen-presenting function of DCs [41]. With this advantage, mature dendritic cell membrane-wrapped nanoparticles can specifically activate T cells due to the peptide/major histocompatibility complex (MHC) complexes on the surface of biomimetic nanoparticles [42]. In addition, costimulatory molecules and adhesion molecules on DC membranes, including integrins, CD44, CD40, and ICAM-3, can facilitate the interaction between T cells and DCs [43]. 

Macrophages can accumulate in the tumor microenvironment owing to some adhesion molecules and specific receptors, such as intercellular adhesion molecule-1, C-C chemokine receptor 2, and vascular cell adhesion molecule-1 (VCAM-1) [43]. In addition, macrophages can achieve active tumor targeting owing to the interaction between α4 integrins on macrophage membranes and VCAM-1 on tumor cell membranes [44].

T cells or cytotoxic T cells are also a subtype of leukocytes that can kill tumor cells. T cells have various properties, such as searching for antigens in the systemic circulation, activating cytolysis by recognizing a single peptide, and producing interferon-γ (IFN-γ, a cytokine with multiple antitumor properties), which make them attractive mediators of antitumor immunity [45,46]. Additionally, cytotoxic T cells can promote tumor cell apoptosis mediated by granule and receptors [47]. In addition, TCR complexes expressed on T cells can specifically bind to tumor-associated antigens with high affinity, which makes T cells more specific in tumor-targeting [48,49]. 

Natural killer (NK) cells play an important role in the recognition and killing of tumor cells owing to their innate capacity to monitor the abnormal expression of stress proteins and MHC-I [50]. Although NK cells lack tumor antigen-specific receptors on their surface, they have some alternative receptors that can recognize tumor cells, such as DNAM-1, NKG2D, and NKp46 [51,52]. Therefore, NK cell membrane-wrapped nanoparticles possess good tumor-targeting ability.

#### 2.1.3. Platelet Membranes

Similar to erythrocytes, platelet cell membrane-wrapped nanoparticles also have a prolonged blood circulation time and are less immunogenic due to their reduced immunogenicity. Firstly, CD47 expressed on the surface of platelets can inhibit the uptake of platelet cell membrane-wrapped nanoparticles by macrophages [53]. Moreover, CD55 and CD59 expressed on the surface of platelet membranes, along with CD47, can avoid immune complement system attack [54,55]. In addition, P-selectin is overexpressed on the surface of platelets, which allows platelet membrane-derived nanoparticles to specifically bind to the CD44 receptors expressed on tumor cells [56]. Thus, nanoparticles can achieve aggressive tumor targeting and long circulation capability through platelet membrane coating.

#### 2.1.4. Cancer Cell Membranes

Cancer cell membrane-camouflaged nanoparticles can enable immune escaping, prolong systemic circulation, and target homotypic tumors owing to a series of membrane proteins expressed by cancer cells [57,58]. CD47 on the tumor cell surface plays an important role in immune escape, especially in 4T1, MDA-MB-231, and MCF-7 [59,60,61,62]. Thomsen–Friedenreich antigen, E-cadherin, Galectin-3, N-cadherin, and epithelial cell adhesion molecules on the surface of tumor cells are essential for homologous targeting and adhesion [63,64,65]. Therefore, the application of cancer cell membranes for nanoparticle surface coating has the unique advantages of inherent immune escaping and homologous targeting and adhesion.

#### 2.1.5. Stem Cell Membranes

Stem cell membranes are easy to isolate and have a various of molecular recognition sites, which can be used in biomimetic nanoparticles [66,67]. Chemokine receptors on mesenchymal stem cell membranes respond to ligand molecules on tumor cells, promoting the migration of mesenchymal stem cells to the tumor [68]. Moreover, P-selectin, E-selectin, and TGF-β expressed on the membrane of mesenchymal stem cells also influence the tumor tropism of mesenchymal stem cells [68,69]. Therefore, mesenchymal stem cell membrane-wrapped nanoparticles have also attracted increasing attention owing to their good biocompatibility, prolonged circulation time, and tumor targeting.

#### 2.1.6. Bacteria Membranes

In the 1890s, William Coley used toxins made from attenuated bacteria to activate the anti-tumor immune system [70]. This is the first report of bacteria being used in cancer immunotherapy. Outer membrane vesicles (OMVs) produced by Gram-negative bacteria are composed of lipid bilayers and inherit various parent bacteria-derived components, such as enzymes, bacteria antigens, adhesins, and a variety of pathogen-associated molecular patterns (PAMPs) [71,72,73]. Among them, bacteria-derived antigens and PAMPs play a vital role in inducing the humoral and cellular anti-tumor immune responses [74,75]. Taking the advantages of OMVs, researchers have attempted to construct bacterial membrane-derived nanoparticles for cancer immunotherapy [76]. 

#### 2.1.7. Extracellular Vesicles

Extracellular vesicles (EVs) are a group of nanoscale membrane-bound vesicles secreted by almost all eukaryotic cells [77,78]. The surface of EVs is rich in a variety of transmembrane proteins, such as ICAM-1, integrin, and tetraspanin, which give EVs the ability to target specific tissues or cells [79]. In addition, compared with traditional drug delivery platforms, EVs have excellent biocompatibility, low immunogenicity, phagocytosis avoidance, controllable biological characteristics, and the potential to cross natural barriers such as the blood–brain barrier [80]. Especially, EVs with immunomodulatory capacities, such as DC-derived EVs, NK-derived EVs, and T cell-derived EVs, can be used as effective therapeutics for cancer immunotherapy [81,82].

#### 2.1.8. Hybrid Cell Membranes

Compared with single-cell membranes, hybrid cell membranes not only retain the physical and chemical characteristics of nanoparticles, but also endow nanoparticles with the biological functions of two or more derived cells, which makes biomimetic cell-derived nanoparticles more attractive [83]. Hybrid cell membrane-camouflaged nanoparticles can enhance the flexibility of nanoparticle functionality and thereby achieve better anti-tumor effects [84,85,86,87].

### 2.2. Fabrication of Biomimetic Cell-Derived Nanoparticles

**Figure 2:**
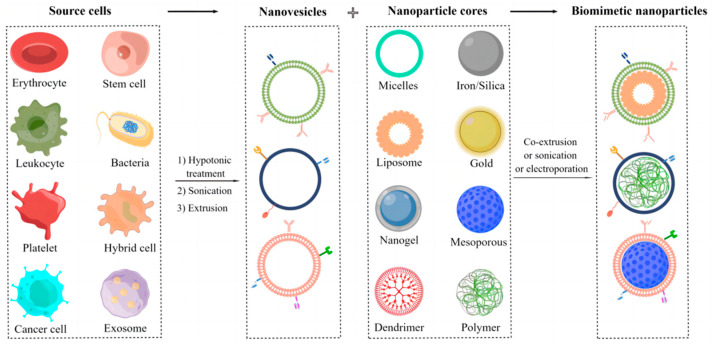
The fabrication of biomimetic cell-derived nanoparticles. By Figdraw.

The fabrication of biomimetic cell-derived nanoparticles mainly includes: (1) isolation and construction of parent cell membrane-derived vesicles and (2) fusion of the parent cell membrane with nanoparticle cores (Figure 2).

#### 2.2.1. Isolation and Preparation of Parent Cell Membrane-Derived Vesicles

The isolation of cell membranes should be gentle in order to obtain biologically active membrane vesicles and usually involves cell lysis and membrane purification. Anucleated cells, such as platelets and erythrocytes, are isolated from whole blood by centrifugation. After that, the collected cells are lysed via repeated freeze–thaw or hypotonic treatment. Purified membranes are obtained by removing soluble proteins by centrifugation. Finally, the purified membranes are extruded through a polycarbonate porous membrane to gain parent cell membrane-derived vesicles [23,54,55]. Bacteria are coated with cell membranes and peptidoglycans, making membrane extraction more difficult. Luckily, Gram-negative bacteria can naturally produce OMVs, which can be directly separated from their culture via ultrafiltration [88,89]. Compared with anucleated cells, the extraction and purification of parent membranes from eukaryotic cells, such as stem cells and leukocytes, is more complicated. First, we have to collect source cells from the tissue, blood, or culture medium. Then, a variety of methods are used for cytolysis, including hypotonic solution treatment, repeated freeze–thaw, and/or mechanical rupture. Additionally, the obtained membranes are purified by discontinuous sucrose gradient centrifugation to remove nuclei, intracellular vesicles, and intracellular biomacromolecules [90,91]. 

#### 2.2.2. Fusion of Parent Cell Membrane-Derived Vesicles with Nanoparticle Cores

Membrane extrusion and ultrasonic and microfluidic electroporation are commonly used methods to facilitate the coating of parent cell membrane-derived vesicles onto nanoparticle cores [2,14]. Mechanical extrusion is one of the commonly used methods reported in the literature. Briefly, the membrane vesicles and nanoparticles are extruded through the polycarbonate porous membrane with progressively smaller pore sizes. The fusion of vesicles and particles is achieved by applying mechanical forces to facilitate the passage of nanoparticles through the lipid bilayer of the membrane vesicles [92]. This approach can largely ensure the bioactivity of membrane proteins. Nevertheless, it is a time-consuming process [93]. Ultrasound is another effective method. When the nanoparticles and membrane vesicles are mixed together, the membranes surrounding the nanoparticles are reassembled by sonication. Compared to the extrusion method, this method is time-saving. However, the parameters of the ultrasound apparatus, including power, frequency, and duration, need to be optimized to guarantee fusion efficiency while avoiding protein inactivation. Microfluidic electroporation can also be utilized for coating different types of nanoparticles. Electromagnetic energy forms holes in cell membranes by a microfluidic chip, thereby promoting membrane vesicles to wrap nanoparticle cores [94]. In this process, the parameters also need to be optimized, such as pulse voltage, flow rate, and duration. The biomimetic cell-derived nanodrugs constructed by this method are completely coated, uniformly distributed, and highly reproducible. However, this technology comes at a high cost. In contrast to nanoparticle-templated membrane coating, Zhang and his colleagues reported a simple method to synthesize cell membrane-wrapped nanogels by cell membrane-templated polymerization [95]. Unlike the previously mentioned approaches, this new strategy uses membrane vesicles to ‘guide’ the growth of nanoparticle cores, thereby surmounting the “coatability” limitation of nanoparticles, as well as adding elevated controllability for the application of biomimetic cell-derived nanoparticles. The key challenge for this technique is how to efficiently and precisely inhibit the polymerization reaction outside the vesicles while maintaining the reaction activity inside the vesicles.

## 3. Application of Biomimetic Cell-Derived Nanoparticles in Cancer Immunotherapy

Considering the outstanding advantages, biomimetic cell-derived nanoparticles have been extensively investigated for cancer immunotherapy. For example, they can be used to deliver immunotherapeutic drugs and immune adjuvants to tumors or improve the effectiveness of cancer immunotherapy by reversing the ITME. In the following, we present the application of various biomimetic cell-derived nanoparticles in ICIs, ACT, cancer vaccines, modulating of the ITME, and combination therapy. 

### 3.1. Immune Checkpoint Inhibitors

Immune checkpoint inhibitors (ICIs), such as programmed death ligand 1/programmed death protein 1 (PD-L1/PD-1) inhibitors, have shown efficacy in anti-cancer immune responses in various cancers [96,97]. However, the clinical use of ICIs is largely limited because only 10 to 30% of cancer patients respond positively to ICIs [98]. To improve the efficiency of ICIs, biomimetic cell-derived nanodrug delivery systems may have great potential. 

Recent studies have found that some cancer cells release a mass of exosomes (named TEXs), which carry a large number of PD-L1s on their surface [99]. Moreover, exosomes expressing PD-L1 can competitively bind and exhaust PD-L1 Ab (aPD-L1), leading to drug resistance to aPD-L1 [100,101]. Thus, suppression of exosomes secreted by tumor cells can be a powerful anti-cancer strategy to improve the effectiveness of current aPD-L1. Herein, Yan and colleagues developed self-adaptive platelet cell membrane-wrapped nanoparticles to enable cascaded delivery of exosome-inhibiting siRNA (siRab) and aPD-L1, resulting in a robust anti-tumor immune response [102]. In their study, siRab effectively suppressed the production of TEXs, alleviated immunosuppression, and enhanced cytotoxic T lymphocyte infiltration to facilitate the on-demand release of aPD-L1 by biomimetic nanoparticles. As a result, the competitive exhaustion of aPD-L1 by TEXs was restrained. Additionally, siRab-mediated reversal of ITME combined with aPD-L1-based immunotherapy induced a robust anti-tumor response and immune memory. 

**Figure 3:**
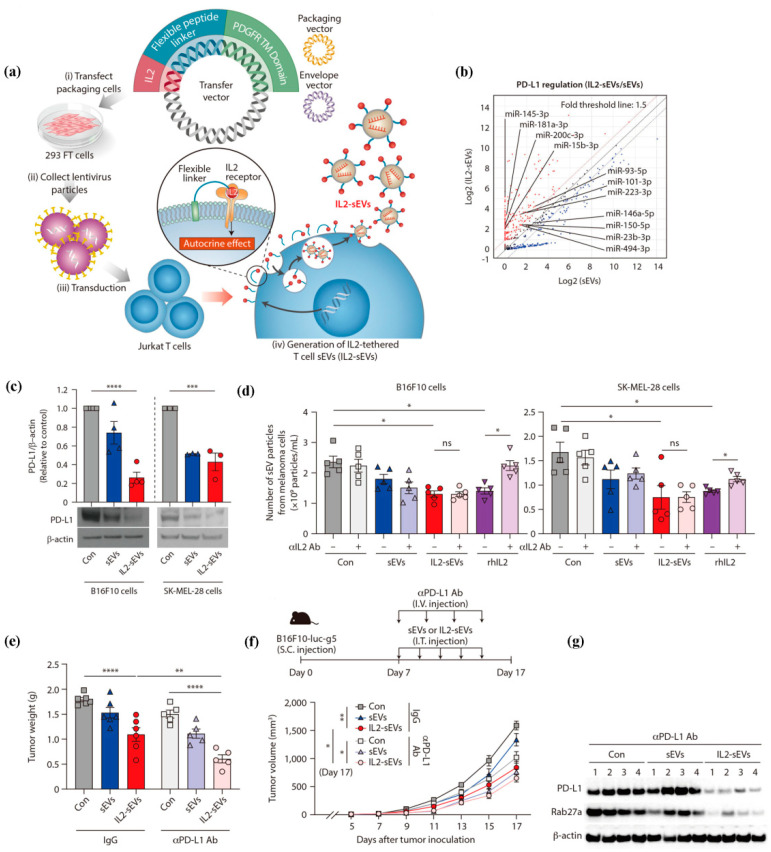
IL2-sEVs to enhance the anti-cancer efficacy of aPD-L1. (**a**) The construction of IL2-sEVs. (**b**) IL2-sEVs altered the miRNA profile of T cell-derived sEVs. (**c**) IL2-sEVs down-regulated PD-L1 levels in melanoma cells. (**d**) IL2-sEVs inhibited sEV secretion. (**e**,**f**) IL2-sEVs enhanced the therapeutic efficacy of aPD-L1. (**g**) Qualitative analysis of PD-L1 in tumor tissues after combined aPD-L1 treatment. ns: no significant, * *p* < 0.05, ** *p* < 0.01, *** *p* < 0.001, **** *p* < 0.0001. Reproduced under the terms of the Creative Commons Attribution-NonCommercial-NoDerivs License [107].

Cancer cells secrete small extracellular vesicles (sEVs) to promote tumor progression, but conversely, immune cells secrete sEVs to prevent tumor progression [103,104,105]. Researchers have found that sEVs secreted by immune cells play a crucial role in anti-cancer immune responses by participating in the interaction between innate immunity and adaptive immunity [103,106]. Herein, Jung and colleagues generated interleukin-2-anchored T cell sEVs (IL2-sEVs) by attaching IL2 to the surface of T cells [107]. In their study, self-stimulation of T cells by IL2 altered the microRNA (miRNA) profile of T cell-derived sEVs. Among them, miR-223-3p and miR-181a-3p distinctly inhibited the sEV secretion and PD-L1 expression in melanoma cells, leading to an elevated immune response. Moreover, IL2-sEVs notably enhanced the therapeutic efficacy of aPD-L1 by reducing PD-L1 expression in vivo (Figure 3).

The accumulation of hypoxia-inducible factor-1α (HIF-1α) in the hypoxic tumor microenvironment has been confirmed to activate the downstream CD73–adenosine (CD73-ADO) pathway and lead to effector T cell exhaustion [108], which is a key disadvantage for the poor clinical efficacy of ICIs treatment [109]. Therefore, it is of great value to alleviate tumor hypoxia and block the CD73-ADO pathway to enhance the therapeutic effect of ICIs. Herein, Yuan and colleagues constructed cancer cell membrane-wrapped and matrix metallopeptidase-sensitive nanoparticles (CSG@B16F10) to co-deliver oxygen-generating agent catalase (CAT) and CD73siRNA, thus alleviating hypoxia and reshaping T cell exhaustion caused by the CD73-ADO pathway [110]. In their study, CAT improved cancer hypoxia by producing abundant endogenous oxygen, while CD73siRNA efficiently inhibited the expression of the target gene, synergistically down-regulating the level of CD73 and promoting the T cell-specific immune response. Moreover, CSG@B16F10 significantly enhanced the tumor immunotherapy efficacy and response rate of aPD-L1 by alleviating hypoxia and reversing the immunosuppressive microenvironment in vivo. 

In another study, Li and colleagues also designed an intelligent biomimetic drug delivery system (mEHGZ) to convert an immunosuppressive microenvironment to an immunoresponsive one, which ultimately enhanced the sensitivity of aPD-L1 immunotherapy [111]. Different from alleviating tumor hypoxia, mEHGZ mainly amplified the generation of immunogenic cell death (ICD) by triggering a cascade reaction for reactive oxygen species (ROS) production, thereby enhancing the sensitivity of immunosuppressed tumors to aPD-L1. After cellular uptake of mEHGZ, a Fenton reaction was triggered by released hemin and glucose oxidase to facilitate ROS generation and increase endoplasmic reticulum stress, which collectively amplified the ICD effect. Furthermore, the induced powerful ICD effect promoted DC maturation and cytotoxic T lymphocyte infiltration, thereby activating the ITME. mEHGZ combined with aPD-L1 significantly inhibited tumor progression and lung metastasis in vivo, indicating that a robust ICD could substantially improve the efficacy of aPD-L1. 

Additionally, a summary of biomimetic cell-derived nanoparticles applied in ICIs is displayed in Table 1.

### 3.2. Adoptive Cellular Immunotherapy

Adoptive cellular immunotherapy (ACT) refers to the in vivo transfusion of immune effector cells activated in vitro to activate the patients’ tumor-specific immune responses or to directly kill tumors. Despite the great potential of ACT to activate the anti-tumor immune responses, its clinical application is severely hampered by disadvantages such as off-target effects, poor infiltration, T-cell exhaustion, in vitro expansion and engineering, severe side effects, and great costs [124,125,126]. Therefore, there is a high demand for the construction of ACT mimics that can conquer the challenges mentioned above using some effective methods, and biomimetic cell-derived nanodrug delivery systems may be a good option.

Kang and colleagues constructed T cell-wrapped nanoparticles (TCMNPs) as a mimicry of CTLs to reactivate the exhausted T cells induced by the ITME [127]. Similar to CTLs, TCMNPs actively targeted tumors via adhesion proteins and directly eliminated tumor cells by releasing dacarbazine and triggering Fas-ligand-induced apoptosis. Unlike CTLs, TCMNPs were free from immunosuppressive cytokines and PD-L1 on cancer cells by blocking TGF-β1 and PD-L1 via TGF-β1 receptors or PD-1 proteins on TCMNPs, which ultimately restored the cytotoxic functions of exhausted T cells. Indeed, the significant inhibitory effect of TCMNPs on melanoma was attributed to the synergistic effect of chemotherapy, TGF-β blocking, and the PD-1/PD-L1 signaling blockade. In another study, Hong and colleagues constructed CD8+ T cell-derived exosomes (TCNVs) that also effectively prevented T-cell exhaustion and reversed ITME in the same way as TCMNPs [128]. Furthermore, TCNVs directly induced apoptosis via the delivery of granzyme B to tumor cells. 

Compared with T cell-based adoptive immunotherapy, NK cells can directly kill tumor cells without causing graft-versus-host disease due to immunocompatibility, making them suitable for a variety of allogeneic and situations [129]. Nevertheless, the number of NK cells in the blood is relatively small, and the cytotoxic effect is relatively weak, which cannot meet the needs of current clinical treatment [130]. Herein, Wu and colleagues designed cancer cell membrane-wrapped magnetic nanoparticles (CM-Fe_3_O_4_@SiO_2_, CMNPs) for stimulating NK cells and enhancing NK cell-based immunotherapy [131]. In their study, CMNPs enhanced the expression of surface-activating hallmark receptors on NK cells and facilitated the production of cytotoxic cytokines such as granzyme and perforin, resulting in enhanced NK cell-mediated immunotherapy. In another study, Gong and colleagues constructed nanobody 7D12-engineered NK cells (7D12-NK92MI) through a glycoengineering approach to improve NK cell-based immunotherapy in EGFR-overexpressing solid tumors [132]. The obtained 7D12-NK92MI exhibited high targeting and affinity to EGFR-positive cancer cells, resulting in good tissue penetration and enhanced secretion of cytotoxic cytokines such as enzyme B, IL-2, and IFN-γ. In addition, 7D12-NK92MI exhibited more significant immunotherapy efficacy in EGFR-positive cancer cell-bearing mice. 

DC-based ACT is another alternative treatment for T cell-mediated immunotherapy [133]. Nevertheless, the ITME can trigger the differentiation of tolerogenic DCs, which in turn induces the apoptosis of CD8+ T cells or activates regulatory T cells, eventually leading to tumor immune escape [134]. Herein, Sun and colleagues constructed smart DCs (iDCs) to restart the cancer immunity cycle by coating IR-797-loaded nanoparticles with mature DC membranes [135]. The obtained iDCs could effectively cross-prime T cells to secrete cytokines and migrate to lymph nodes to activate the initial T cell. Then, the activated T cells up-regulated the levels of heat shock proteins in cancer cells, thus making tumor cells more susceptive to heat stress. Moreover, radiation therapy with mild photothermal therapy effectively killed cancer cells and further induced the immune responses by releasing ROS as well as other immunomodulators. Consequently, the dying cancer cells combined with activated immune cells synergistically triggered powerful ICD and restarted the everlasting cancer immunity cycle, exhibiting a synergistic anticancer effect. 

Additionally, a summary of biomimetic cell-derived nanoparticles applied in ACT is displayed in Table 2.

### 3.3. Cancer Vaccines

Cancer vaccines, including DNA vaccines, mRNA vaccines, peptides, tumor-specific proteins, tumor cells/lysates, and personalized vaccines coated with tumor cell membranes, can activate the anti-cancer immune responses by presenting tumor antigens and adjuvants to the host immune system [139,140,141]. Nevertheless, challenges in vaccine manufacturing, genetic heterogeneity among patients, limitations of immune response identification, and the disadvantages of the ITME seriously hampered the clinical application of cancer vaccines [139,142]. Therefore, nanovaccines as an alternative strategy in highly effective immunotherapy are attracting more and more attention [142,143,144].

Inspired by in situ vaccines, Xiong and colleagues constructed a personalized vaccine (R@P-IM) by camouflaging R837-loaded nanoparticles with calcinetin (CRT)-expressed cancer cell membranes containing tumor-associated antigens [145]. The engineered tumor-associated antigens constructed by ICD induction in vitro retained a complete antigen array while alleviating the acute systemic toxicity of chemotherapy in vivo. Meanwhile, the CRT expressed on the surface of the nanovaccine facilitated the internalization of the nanovaccine by DCs, consequently enhancing the immune responses. Subsequently, adjuvant R837 released from the nanovaccine excited toll-like receptor 7 (TLR7), which further activated DCs. In addition, the nanovaccine activated memory T cells for long-term protection, exhibiting a satisfactory immunotherapeutic efficacy for cancer therapy and prevention. 

Although cancer cell membrane-derived nanovaccines can stimulate multiantigenic immunities with enhanced anticancer efficacy, once these nanovaccines enter the blood capillary via lymphatic capillaries, the adjuvants leakage usually triggers severe systemic inflammation. In order to avoid this high risk, there is an urgent need to construct biomimetic nanovaccines based on nanoparticle cores with weak or no immune stimulatory effects. Li and colleagues constructed a biomimetic nanovaccine (CCM@(PSiNPs@Au)) based on weak-immunostimulatory silicon@Au nanoparticles [146]. In their study, CCM@(PSiNPs@Au) with a weak immune stimulatory effect still efficiently delivered cancer cell membranes into DCs and triggered DC maturation, thereby leading to the activation of the downstream anti-tumor immune responses. Moreover, CCM@(PSiNPs@Au), as a photothermal therapeutic agent, combined with immunotherapies exhibited a satisfactory immunotherapeutic efficacy for cancer occurrence and development by activating the anti-cancer immune responses and reversing the ITME in vivo. 

**Figure 4:**
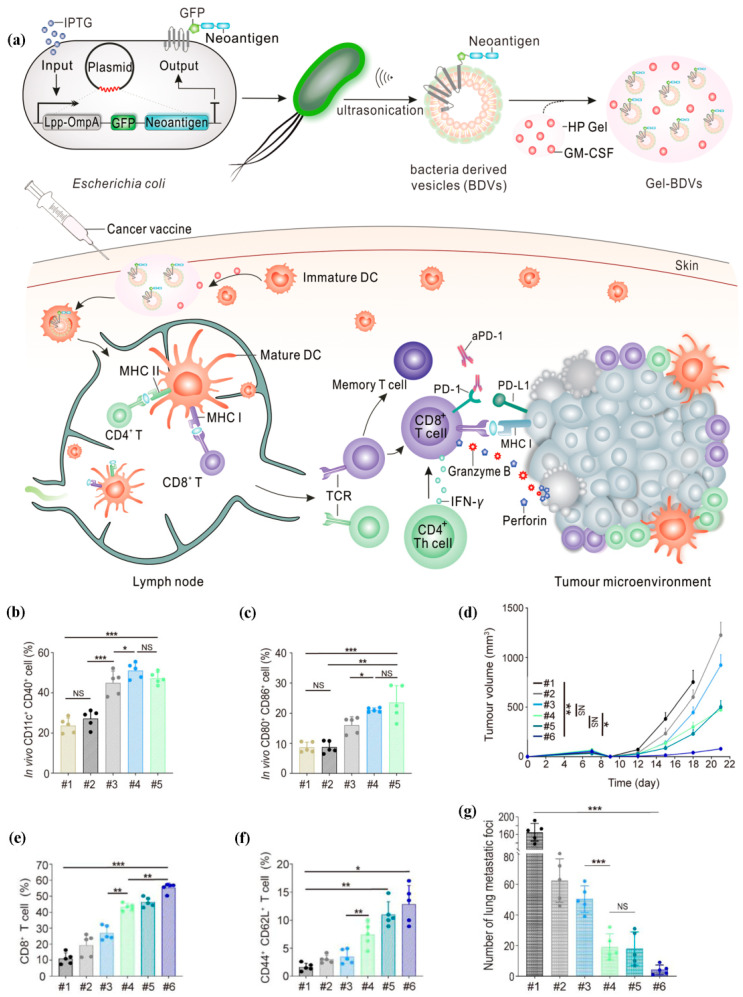
BDVs-Neo vaccine combined with aPD-L1 for immunotherapy. (**a**) The preparation of BDVs-Neo vaccine. (**b**,**c**) DC activity and maturation induced by BDVs-Neo in lymph nodes. (**d**) BDVs-Neo combined with aPD-L1 for anti-tumor recurrence. (**e**,**f**) Synergistic mechanism of BDVs-Neo in combination with aPD-1. (**g**) BDVs-Neo vaccine combined with aPD-L1 for lung metastasis. (#1) Gel-PBS, (#2) Gel-Blank BDVs, (#3) Gel-Normal-M33-M47 BDVs, (#4) Gel-Mutation-M33-M47 BDVs, (#5) aPD-1, (#6) Gel-Mutation-M33-M47 BDVs + aPD-1. NS: no significant, * *p* < 0.05, ** *p* < 0.01, *** *p* < 0.001. Reproduced under the terms of the Creative Commons Attribution-NonCommercial-NoDerivs License [150].

Neoantigens are abnormal proteins generated only by tumor cells, which are called tumor-specific antigens [147]. Therefore, neoantigens can prevent “off-target” destruction to normal tissues and are not affected by central or peripheral tolerance, making them promising candidate antigens for personalized cancer immunotherapies [148,149]. Meng and colleagues engineered bacteria to fabricate fusion neoantigens and constructed bacteria-derived neoantigen-bearing vesicles (BDVs-Neo) as an individualized cancer vaccine to trigger the systemic anti-tumor immune responses [150]. Then, BDVs-Neo and granulocyte-macrophage colony-stimulating factor (GM-CSF, an adjuvant) were injected subcutaneously within temperature-sensitive hydrogels. When combined with aPD-1, the sustained release of GM-CSF and BDVS-lipopolysaccharide (LPS) in hydrogels recruited DCs and provided long-term memory immunity by intensively enhancing the proliferation and activation of tumor-infiltrating lymphocytes (TILs) and clonal expansion of memory T cells (Figure 4).

Herpesvirus, a major human pathogen, can bind specifically to cancer cells by identifying tumor-associated antigens on the surface of tumor cells, and trigger strong and long-lasting anti-cancer immune responses by inducing mitochondrial DNA (mtDNA) stress [151]. In addition, Mn^2+^ is released from organelles and accumulates in the cytoplasm during herpesvirus infection, which promotes the antiviral innate immune responses by increasing the sensitivity of cGAS to mtDNA and facilitating STING activation [152,153]. Herein, encouraged by the strong innate immunity triggered by herpesvirus, engineering erythrocyte membrane-wrapped DNAzyme-loaded nanoparticles (Vir-ZM@TD) were constructed for cancer immunotherapy by simulating the structure and infection processes of herpesvirus [154]. Vir-ZM@TD not only effectively prolonged the blood circulation time of nanoparticles, but also closely mimicked a series of herpesvirus infection processes, including specific tumor targeting, effective endosomal escape mediated by membrane fusion, mitochondrial DNA stress triggered by transcription factor A, and Mn^2+^ release from organelles into the cytoplasm. Thus, the anti-cancer immune response triggered by the cGAS-STING innate pathway was effectively activated, and the complete tumor regression was about 68%.

Additionally, a summary of biomimetic cell-derived nanoparticles applied in cancer vaccines is displayed in Table 3.

### 3.4. Modulating the Immunosuppressive Tumor Microenvironment

**Figure 5:**
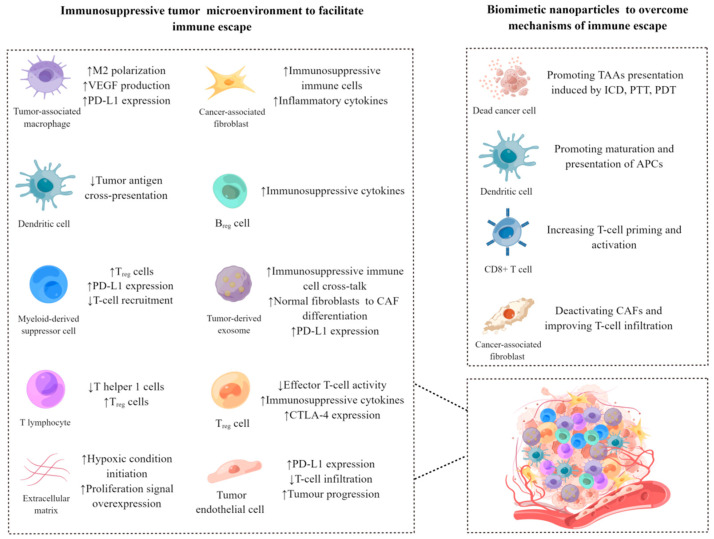
The immunosuppressive tumor microenvironment (ITME) and biomimetic cell-derived nanoparticles for reversing the ITME. By Figdraw.

The immunosuppressive tumor microenvironment (ITME) supports tumor escape from immune surveillance and is a major obstacle to immunotherapy [165,166]. Multiple complex factors contribute to the ITME (Figure 5). The low immunogenicity of tumors impede recognition by the immune system [167]. A variety of immunosuppressive cells and cytokines hinder anti-tumor immune responses via different pathways [168]. The extracellular matrix of the tumor prevents the infiltration of anti-tumor immune cells [169]. The hypoxia and abnormal metabolic activities of tumors are conducive to the immune escape of tumors [170,171,172]. Reversing the ITME is beneficial to the recruitment and activation of anti-tumor immune cells, which can promote immunotherapy, and biomimetic cell-derived nanodrug delivery systems may be a good choice.

Tumor-associated macrophages (TAMs) are an important part of the ITME [173]. TAMs can be polarized into M2-like phenotypes, which trigger the occurrence, progression, and recurrence of tumors [174]. Fortunately, TAMs have a certain degree of plasticity and can be converted to M1 type to inhibit tumor growth [175]. Wei and colleagues constructed a bacteria–nanoparticles complex (Ec-PR848) for TAM polarization and enhanced immunotherapy [176]. This smart biomimetic nanoparticle platform greatly repolarized M2-type macrophages towards M1-type macrophages, resulting in the generation of TNF-α, an increased expression of IL-6, as well as the activation of anti-cancer immunity. When supplemented with PLGA-DOX-triggered ICD, Ec-PR848 further weakened the degree of tumor immunosuppression and subsequently induced a strong anticancer immune response. In another study, Rao and colleagues show that SIRPα variants-overexpressed cancer cell membrane-wrapped magnetic nanoparticles (gCM-MNs) could effectively polarize M2 macrophages to M1 macrophages by the magnetic nanoparticle cores, facilitating macrophages to phagocytose cancer cells and enhancing anticancer T-cell immunity [177]. 

In addition to M2-type TAMs, the presence of tumor hypoxia is also significantly associated with the ITME [178]. Research shows that remodulating tumor hypoxia can facilitate cancer immunotherapy by stimulating anti-tumor T cells and NK cells, reducing macrophage recruitment and PD-L1 on cancer cells, and maintaining M1-TAMs polarization [179,180]. Herein, Wang and colleagues constructed a biomimetic drug delivery system (V(Hb)@DOX) for ITME remodulation by co-delivering oxygen and DOX using erythrocyte membrane camouflaged amphiphilic PCL nanoparticles [181]. The Hb moiety of V(Hb)@DOX effectively killed cancer cells by specifically targeting M2-type TAMs via the CD163 receptor, while the O_2_ released by Hb relieved cancer hypoxia and further enhanced the anti-cancer immunity by reducing the recruitment of M2-type TAMs, which synergistically reversed the ITME by down-regulating the expression of PD-L1 on cancer cells, reducing levels of immunosuppressive cytokines, increasing immunostimulant IFN-γ, enhancing CTL response, and inducing a robust memory immunity.

CAFs, as the main components of tumor stroma, provide a tremendous energy supply to tumor cells via the glycolytic pathway [182]. In addition, lactate produced by CAFs and cancer cells via glycolysis often results in the ITME [183,184]. Therefore, metabolic reprogramming by destroying the metabolic networks between CAFs and cancer cells may be a key to enhancing cancer immunotherapy. Zang and colleagues constructed a biomimetic nano-delivery system by using hybrid membranes of activated fibroblasts and cancer cells to coat solid lipid nanoparticles containing the glycolytic inhibitor PFK15 and chemotherapeutic drug PTX [185]. The obtained biomimetic nanoparticles (PTX/PFK15-SLN@ [4T1-3T3] NPs) possessed homologous targeting towards both CAFs and tumor cells. The encapsulated PFK15 effectively blocked glycolysis in both CAFs and tumor cells, thereby cutting off the energy supply of CAFs to tumor cells. Moreover, PTX/PFK15-SLN@ [4T1-3T3] significantly reduced lactate production in CAFs and cancer cells, thus reversing the ITME and thereby enhancing anti-cancer immunity.

Different from apoptosis which is generally considered to be an immune tolerance process, pyroptosis is a highly inflammatory programmed cell death (PCD) triggered by caspase-3, demonstrating a good opportunity to alleviate the ITME and facilitate the systemic immune responses [186,187]. Zhao and colleagues constructed a biomimetic nano-delivery system (BNP) by using a breast cancer membrane to coat PLGA nanoparticles containing indocyanine green (ICG) and decitabine (DCT) for photo-triggered cancer pyroptosis and cancer immunotherapy [188]. In their study, ICG induced a sharp increase in cytosolic Ca^2+^ concentration by NIR, which promoted the release of cytochrome c and subsequently activated caspase-3. DCT synergistically up-regulated the expression of gasdermin E by inhibiting DNA methylation, thereby enhancing the cleavage of gasdermin E by caspase-3 and causing robust cell pyroptosis. In particular, the cell pyroptosis triggered DC maturation, activated T cells, and exhibited powerful effects on primary and distant tumor immunotherapy. 

Additionally, a summary of biomimetic cell-derived nanoparticles applied in modulating the ITME is displayed in Table 4.

### 3.5. Combination Therapy

Cancer is a complex disease with a variety of intricate factors, which makes it more difficult to treat different types of cancer with monotherapy [200]. It is suggested that combination therapy is a new trend for cancer treatment in the future. The combination of chemotherapy, radiotherapy, PDT, or other therapies with immunotherapy is the main combination treatment modality [201]. Additionally, biomimetic cell-derived nanocarriers play an important role in cancer combination therapy because of their unique superiorities.

**Figure 6:**
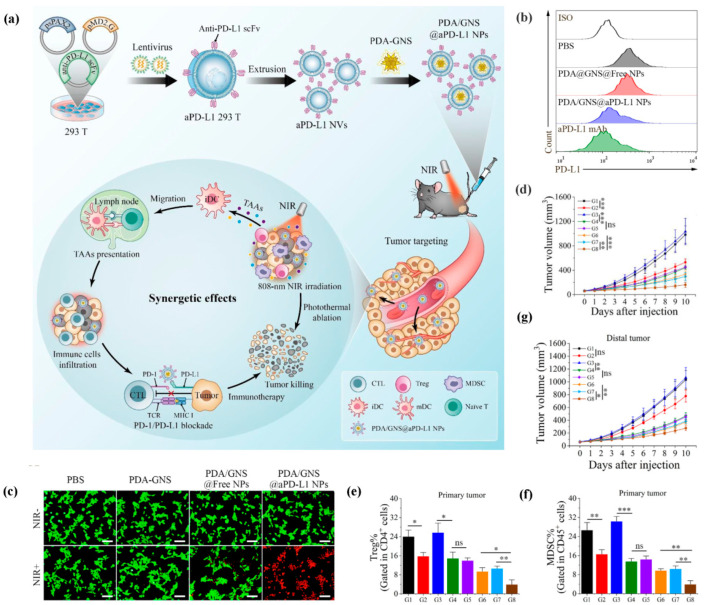
PDA/GNS@aPD-L1 nanoparticles for cancer immunotherapy by combining PTT with PD-1/PD-L1 blockade. (**a**) The preparation of PDA/GNS@aPD-L1. (**b**) PDA/GNS@aPD-L1 binding capability for PD-L1-expressing cells. (**c**) PTT effects of PDA/GNS@aPD-L1. Scale bar: 100 μm. (**d**) Anti-cancer effect of PDA/GNS@aPD-L1 in primary tumor. (**e**,**f**) PDA/GNS@aPD-L1 reversed the ITME by decreasing the number of Treg and MDSC cells at the tumor site. (**g**) Anti-cancer effect of PDA/GNS@aPD-L1 in distal tumor. G1, PBS group; G2, PDA-GNS + NIR group; G3, PDA/GNS@Free NPs group; G4, PDA/GNS@aPD-L1 NPs group; G5, aPD-L1 mAb group; G6, aPD-L1 NVs + PDA-GNS + NIR group; G7, aPD-L1 mAb + PDA-GNS + NIR group; G8, PDA/GNS@aPD-L1 NPs + NIR group. ns: no significant, * *p* ≤ 0.05, ** *p* ≤ 0.01, *** *p* ≤ 0.001. Reproduced under the terms of the CC BY-NC-ND license [202].

Xiao and colleagues constructed biomimetic nanoparticles (PDA/GNS@aPD-L1) to combine PTT with PD-1/PD-L1 blockade for synergistic tumor inhibition in colorectal cancer [202]. Firstly, polydopamine-modified gold nanoparticles (PDA-GNS) were prepared as nanoparticle cores. Subsequently, cell membranes extracted from anti-PD-L1 scFv-engineered HEK 293T cells were applied to camouflage these PDA-GNs cores. Anti-PD-L1 scFv on the biomimetic nanoparticles not only blocked the PD-1/PD-L1 signal, but also promoted the aggregation of PDA-GNs at the tumor site. What is more, PDA-GNs-induced PTT triggered the release of tumor-associated antigens and reversed the ITME, which thereby greatly improved the outcome of ICI immunotherapy (Figure 6). 

Lu and colleagues constructed tumor cells and DCs dual active-targeting biomimetic nanoparticles (AMR-MOF@AuPt) as a sono-immunotherapeutic nanoplatform for multimodal therapies [203]. In their study, the sono-responsive nanometal organic frameworks (MOFs) were successfully coated by engineering cancer cell membranes displaying anti-DEC205 antibodies. Anti-DEC205-anchored cell membranes directly targeted and activated DCs to facilitate tumor antigen cross-presentation, thereby triggering a cascade of T cell immune responses. More interestingly, AMR-MOF@AuPt-triggered SDT observably inhibited tumor growth through ROS and induced ICD of cancer cells, which further activated and proliferated cytotoxic T cells. This design allowed for multicellular engagement between tumor cells, DCs, and T cells, which thereby induced a robust systemic and long-term immunity to inhibit tumor relapse and distant metastasis. 

Wang and colleagues constructed a PLGA-based biomimetic nano-delivery system (AFT/2-BP@PLGA@MD) which integrated small molecule targeted therapy with immunotherapy [204]. In this platform, the palmitoylation inhibitor 2-bromopalmitic acid (2-BP) was encapsulated in PLGA nanoparticles to enhance the treatment efficacy of afatinib against EGFR-TKIs non-sensitive cancer cells. Then, drug-loaded PLGA nanoparticles were wrapped with cancer cell membranes modified by a D-peptide antagonist (DPPA-1) to block PD-1/PD-L1. In their study, AFT/2-BP@PLGA@MD observably inhibited cancer occurrence and progression by directly killing cancer cells and triggering systemic T cell responses. Their findings indicated that small molecule targeted therapy combined with immunotherapy could break through their respective limitations and obtain stronger anti-cancer efficacy. 

Li and colleagues constructed a multifunctional biomimetic delivery system (AuDRM) to realize the synergistic effect of starvation/PTT/immunotherapy [205]. Briefly, Au NPs were developed in the mesopores of mesoporous silica and then the obtained nanocomplexes were camouflaged with pH-responsive hybrid cancer cell membrane fragments. When AuDRM nanoparticles were efficiently accumulated at the tumor site, the pH-responsive membranes fell off, thereby facilitating the exposed Au NPs for starvation treatment and the released immunostimulant R837 for enhanced immunotherapy. Importantly, upon irradiation, Au NPs caused PTT and then triggered ICD. The resulting immunogenicity, together with the immunostimulant R837 released by AuDRM, stimulated stronger immune responses for cancer therapy.

**Figure 7:**
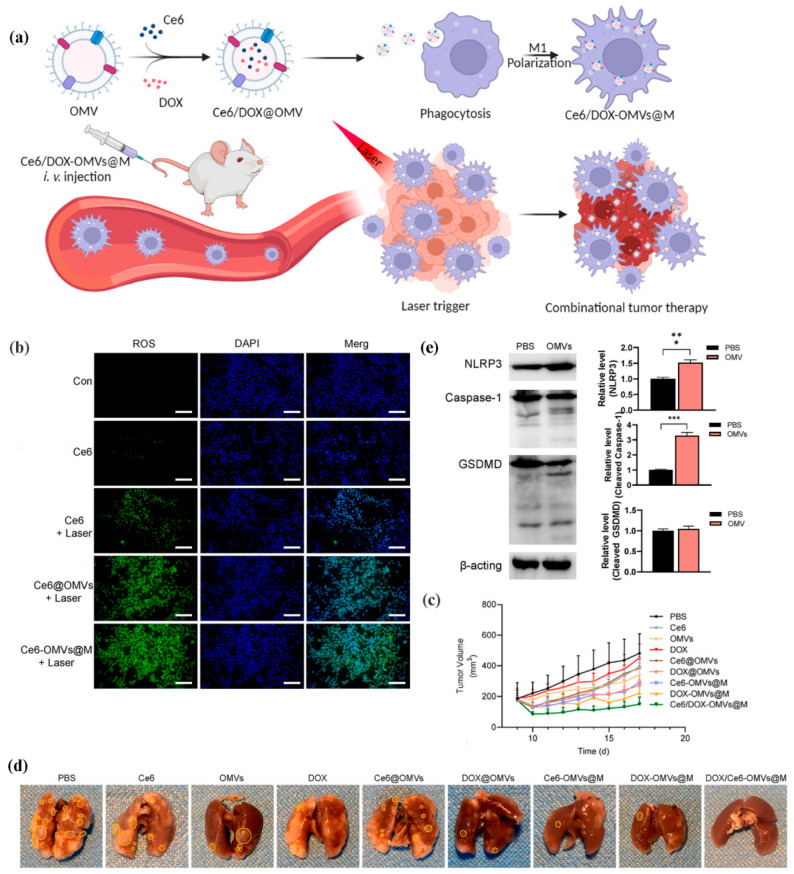
Ce6/DOX-OMVs@M for combined tumor chemo/photodynamic/immunotherapy. (**a**) The preparation of Ce6/DOX-OMVs@M. (**b**) Generation of ROS by Ce6/DOX-OMVs@M in 4T1 cells. Scale bar: 100 μm. (**c**) Anti-tumor effect of Ce6/DOX-OMVs@M. (**d**) Ce6/DOX-OMVs@M for lung metastasis. (**e**) OMVs activated pyroptosis in tumors in vivo. * *p* < 0.05, ** *p* < 0.01, *** *p* < 0.001. Reproduced under the terms of the CC BY-NC-ND license [206].

To improve the safety and efficacy of OMVs in cancer treatment, Li and colleagues loaded OMVs into macrophages and used OMVs as platforms for co-delivery of DOX and Ce6 for combined tumor chemo/photodynamic/immunotherapy [206]. In their study, OMVs effectively polarized M2 macrophages to M1 macrophages and activated pyroptosis-related pathways, thereby enhancing antitumor immunity. The developed Ce6/DOX-OMVs@M demonstrated high safety and exhibited satisfactory immunotherapeutic efficacy for tumor ablation and metastasis (Figure 7). 

Additionally, a summary of biomimetic cell-derived nanoparticles applied in combination therapy is displayed in Table 5.

## 4. Conclusions and Challenges

With new advances in the construction of nano-based drug delivery systems, researchers have successfully developed various of biomimetic cell-derived nanoparticles. This review highlights the recent developments in biomimetic cell-derived nanoparticles for cancer immunotherapy. 

**Figure 8:**
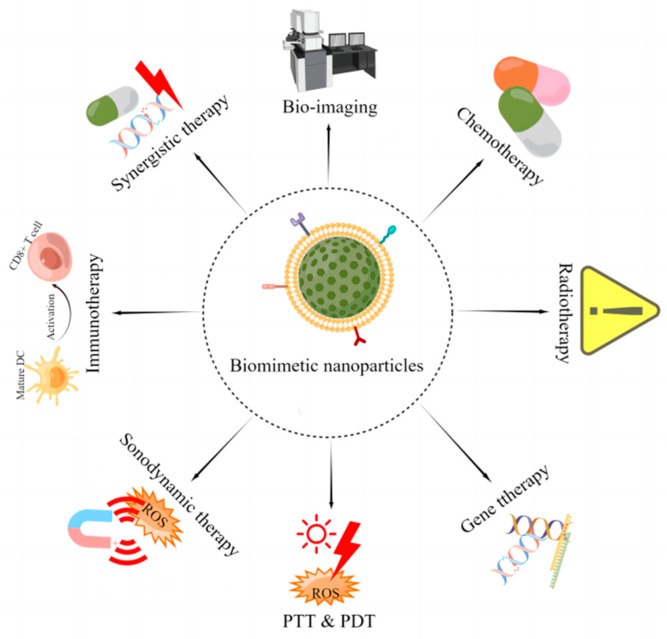
Biomimetic cell-derived nanoparticles for cancer bio-imaging, prevention, diagnosis, and treatment. By Figdraw.

Benefiting from ideal characteristics, biomimetic cell-derived nanoparticles can effectively deliver immunotherapeutic agents and/or immunostimulants to tumor sites, reverse the ITME to an immune-supportive one, trigger ICD, induce the release of tumor-associated antigens, facilitate DCs maturation and tumor antigen presentation, as well as activate T and NK cells, which can observably promote the immune responses and improve the efficacy of cancer immunotherapy. The critical analysis of abundant evidence acquired from existing research suggests that biomimetic cell-derived nanoparticles with cell membrane camouflage can be effectively used for cancer bio-imaging, prevention, diagnosis, and treatment (Figure 8). 

Although the advantages of biomimetic cell-derived nanoparticles have promoted the development of cancer immunotherapy, many challenges still need to be overcome before their clinical transformation. 

Firstly, the anti-cancer efficacy and long-term safety of these emerging biomimetic nanoparticles have not been fully established in humans, which may be one of the key scientific questions for scientists to address in the future. Moreover, the pharmaceutical spectrum of these biomimetic nanoparticles can be further expanded to the delivery of different types of drugs to manage other types of diseases. 

Secondly, the isolation of the cell membrane mainly includes cell lysis, cell contents removal, and purification. The removal of cytoplasmic components by differential centrifugation in the current studies may not completely remove all cytoplasmic components and may instead lead to the loss of membrane fragments. In addition, gentle extraction is required because functional proteins on the cell surface are very susceptible to denaturation and inactivation. How to extract high-purity and intact cell membranes to make sure that the cell membranes can completely inherit the biological functions of the source cells deserves further study. 

Thirdly, the extrusion method generally used to prepare biomimetic nanoparticles is time-consuming, inefficient, and difficult to scale up in industrial production. Moreover, the uncontrollable stability of the production process and the high cost of the manufacturing method are also significant challenges. Therefore, the high-quality control and large-scale preparation of biomimetic nanoparticles are urgent problems to be solved.

Fourthly, current complexity and reproducibility issues are also major challenges associated with biomimetic nanoparticles. In general, the more complicated the preparation processes, the worse the reproducibility. Researchers should prioritize the scale-up of biomimetic nanoparticles in the early stages of nanoparticle design, and the preparation methods and technologies used to construct biomimetic nanoparticles should be simple, reliable, and mature enough to meet the requirements of large-scale production. In addition, the introduction of new preparation methods, such as microfluidic electroporation, is also a future direction. Moreover, surface membrane components are known to play a key role in the design of biomimetic nanoparticles, but the guidelines on which kind of cell membrane may work better for a specific application are not available. According to the current literatures, immune cell membranes and cancer cell membranes are mainly used to fabricate biomimetic nanovaccines for cancer immunotherapy, EVs are mainly used to treat neurological diseases or deliver nucleic acid drugs to treat genetic diseases and cancer, and platelet membrane-derived nanoparticles can effectively locate atherosclerotic plaques for atherosclerosis detection.

Lastly, tumor heterogeneity is a common challenge in the successful treatment of cancer. In cancer immunotherapy, the response of the immune system against antigen-positive cells can lead to selective pressure towards antigen-negative cells, which is a common cause of cancer recurrence. Combined with high-level techniques, developing some individualized biomimetic nanomedicines and improving their efficacy in cancer immunotherapy may be effective problem-solving strategies. 

Although the clinical translation of biomimetic cell-derived nanoparticles faces many challenges, it is undeniable that biomimetic cell-derived nanoparticles have unique advantages and great potential in cancer immunotherapy. 

## Figures and Tables

**Table 1:** Application of biomimetic cell-derived nanoparticles in ICIs.

Cell Membrane	Core Nanoparticle	Characteristics of the Core Nanoparticle	Drug/Imaging Agent Encapsulated	Cancer Models and the Reason for Selecting Each Cancer Cell Line	Biomedical Application	Ref.
Platelet membrane	Rab27 siRNA (siRab) polycationic nanocomplexes and aPD-L1 nanogels	Desired siRNA encapsulation and cell-transfection abilities, good biocompatibility, rapid clearance by macrophages	siRab and aPD-L1	B16F10 cells, leading tumor	siRab silenced Rab27a, inhibited exosome secretion, relieved immunosuppression, and sensitized ICI.	[102]
T cell-derived sEVs	--	--	Membrane-bound IL2	B16F10 and B16F10-luc-g5 cells, leading tumor	IL2-sEVs inhibited sEV secretion and PD-L1 expression by cancer cells through altering the miRNA profile of T cell-derived sEVs, and, consequently, sensitized ICI.	[107]
Cancer cell membrane	Gelatin nanoparticles	Good biocompatibility, matrix metallopeptidase (MMP)-sensitive, low passive targeting efficiency, rapid clearance by macrophages	CAT and CD73 siRNA	B16F10 cells, homologous targeting	CAT relieved tumor hypoxia, while CD73 siRNA silenced CD73 protein and promoted T cell-specific immune response, which synergically sensitized ICI.	[110]
Calreticulin over-expressed cancer cell membrane	Zeolitic imidazolate framework (ZIF-8) nanoparticles	Large surface area, pH-induced biodegradability, low passive targeting efficiency	Epirubicin (EPI), glucose oxidase (Gox) and hemin	4T1 cells, homologous targeting	The biomimetic delivery system displayed an amplified ICD and activated the tumor immune microenvironment, boosting the therapeutic efficacy of ICI.	[111]
Cancer cell membrane	Thermosensitive lipid nanoparticles	Thermosensitive, controlled drug release, good biocompatibility, low passive targeting efficiency	PD-1/PD-L1 inhibitor BMS202 and IR780	4T1 and MCF-7 cells, homologous targeting, and multiple mouse models	The biomimetic delivery system inhibited cancer-associated fibroblasts (CAFs), increased the penetration of TILs, blocked the pathway of PD-1/PD-L1, and then sensitized ICI.	[112]
Bacterial outer membrane	--	--	Membrane-bound PD1 ectodomain	B16F10 and CT26 cells, multiple mouse models	The biomimetic delivery system could effectively block the PD-1/PD-L1 pathway, protect T cells, and sensitize ICI.	[113]
HEK 293T cell membrane	IR820-dihydroartemisinin (DHA) complexes	pH-sensitive, excellent biodegradability, sonodynamic therapy (SDT) nanoplatform, low passive targeting efficiency, rapid clearance by macrophages	IR820 and DHA	Hep1–6 cells, one of the major tumors	DHA-mediated chemo-dynamic therapy and IR820-induced sonodynamic therapy synergistically achieved a robust ICD effect and sensitized ICI.	[114]
Erythrocyte membrane	Liposomes	Good biocompatibility and biodegradability, pH-sensitive, low passive targeting efficiency, rapid clearance by macrophages	Paclitaxel (PTX)	PC-3 cells, one of the major tumors	The biomimetic delivery system reinforced active tumor-targeting behavior and sensitized ICI.	[115]
Cancer cell membrane	Mesoporous organosilica nanoparticles (MONs)	Good biocompatibility, large surface area, X-ray-induced biodegradability, low passive targeting efficiency	Doxorubicin (DOX)	4T1 cells, homologous targeting	The biomimetic delivery system exhibited enhanced DOX-mediated ICD and sensitized ICI.	[116]
Cancer cell membrane	Polymeric nanoparticles	Redox-responsive, good biocompatibility, low passive targeting efficiency	Toyocamycin (Toy) and generation 3 phosphorus dendrimer-copper (II) complexes (1G3-Cu)	B16F10 cells, homologous targeting	Toy-triggered amplification of ER stress and 1G3-Cu-mediated mitochondrial dysfunction synergistically induced significant ICD, which thereby sensitized ICI.	[117]
aPD-L1 over-expressed HEK 293T cell membrane anchored with a cleavable peptide by matrix metallopeptidase 2 (MMP2)	Barium titanate (BTO) nanoparticles	Piezocatalysis materials, generating a cascade of redox reactions under US, rapid clearance by macrophages	BTO and membrane-bound aPD-L1	B16F10 cells, leading tumor	The membrane-bound aPD-L1 could effectively block the PD-1/PD-L1 pathway, while the encapsulated BTO could achieve a piezocatalysis effect and induce tumor antigen release, which synergistically improved ICI therapy.	[118]
Cancer cell-derived EVs with PD-L1 knockout	Liposomes	Good biocompatibility and biodegradability, low passive targeting efficiency, rapid clearance by macrophages	DOX	4T1 cells, homologous targeting	The biomimetic delivery system up-regulated the expression of PD-L1 in the tumor and amplified the ICD mediated by DOX, which thereby sensitized ICI.	[119]
Mature dendritic cell membrane	Redox-responsive nanoparticles	Redox-responsive, low passive targeting efficiency, rapid clearance by macrophages	Oxaliplatin (OXA) prodrugs	CT26 cells, homologous targeting	The biomimetic delivery system effectively sensitized the TME to ICI by immunogenic chemotherapy and tumor antigen-specific immunotherapy.	[120]
Platelet membrane	Magnetic nanoparticles	Good biocompatibility, effective ferroptosis, magnetic targeting, rapid clearance by macrophages	Sulfasalazine (SAS)	4T1 cells, classic tumor model	The biomimetic delivery system-mediated ferroptosis elicited an effective immune response and sensitized ICI.	[121]
Erythrocyte membrane	Oligomeric Au (I)-PMIV complexes	Good biocompatibility, effective, self-assembling, low passive targeting efficiency, rapid clearance by macrophages	PMIV (a peptide that can degrade MDM2/MDMX)	NCI-H1650 cells, LUAD-patient-derived xenograft mice model, and LUAD-PDX mice model, multiple mouse models	The biomimetic delivery system potently restored p53 and p73, and sensitized ICI.	[122]
HEK 293T cell membrane expressing CD64	--	--	Membrane-bound aPD-L1 and cyclophosphamide	B16F10-luci cells, leading tumor	The biomimetic delivery system restrained the Tregs and invigorated Ki67+CD8+ T cells, then improved ICI therapy.	[123]

**Table 2:** Application of biomimetic cell-derived nanoparticles in ACT.

Cell Membrane	Core Nanoparticle	Characteristics of the Core Nanoparticle	Drug/Imaging Agent Encapsulated	Cancer Models and the Reason for Selecting Each Cancer Cell Line	Biomedical Application	Ref.
T cell membrane	Poly(lactic-co-glycolic) acid (PLGA) nanoparticles	Good biocompatibility and biodegradability, low passive targeting efficiency, rapid clearance by macrophages	Dacarbazine (DTIC)	B16F10 cells, DTIC for metastatic melanoma treatment	The biomimetic delivery system could induce Fas-ligand-mediated apoptosis and scavenge TGF-β1 and PD-L1 to restore the function of CTLs.	[127]
T cell membrane over-expressing PD-1, TGF-β receptor, and granzyme B	--	--	Granzyme B	LLC cancer cells, immunotherapy for lung carcinoma	The biomimetic delivery system prevented CD8+ T cell exhaustion mediated by PD-L1/TGF-β, and promoted tumor cell apoptosis via granzyme B.	[128]
Cancer cell membrane	Magnetic nanoparticles	Good biocompatibility, magnetic targeting, rapid clearance by macrophages	--	HepG2 and A375 cells, homologous targeting, and multiple mouse models	The biomimetic delivery system effectively activated NK cells and enhanced NK cell-based ACT through the enhanced secretion of soluble cytotoxic effectors.	[131]
NK92MI cell membrane equipped with the anti-EGFR nanobody 7D12	--	--	--	EGFR^positive^ cancer cell lines, including LoVo, MDA-MB-468, A549, and A431 cells, 7D12 for specifically recognizing EGFR^positive^ cancer cells	The biomimetic delivery system exhibited high targeting for EGFR-positive cancer cells, caused enhanced secretion of cytotoxic effectors, and improved NK cell-based ACT.	[132]
Mature DC membrane	Polymeric nanoparticles	Good biocompatibility and biodegradability, low passive targeting efficiency, rapid clearance by macrophages	IR-797	4T1 cells, specific recognition between tumor specific antigens and tumor cells	Mature DC membrane facilitated effective cross-priming of T cells, recruited T cells, and produced immunostimulatory cytokines, while mild PTT mediated by IR-797 induced a greater degree of tumoricidal effect, which consequently amplified ICD and reinitiated the self-sustaining cycle of cancer immune responses.	[135]
Cancer cell membrane	MnOx nanoparticles functionalized with anti-CD3/CD28 mAbs	Good biocompatibility, low passive targeting efficiency, rapid clearance by macrophages	anti-CD3/CD28 mAbs	B16F10 cells, homologous targeting	The biomimetic delivery system not only efficiently promoted the expansion and activation of CD8+ T cells and DCs but also reversed the immunosuppressive microenvironment to promote T cell survival.	[136]
NK92MI cell membrane	Anti-CD56 antibodies modified Fe_3_O_4_ nanoparticles	Good biocompatibility, magnetic targeting, rapid clearance by macrophages	--	K562, B16F10, and H22 cells, NK92 cell possessing a broader-spectrum anti-cancer activity	The biomimetic delivery system up-regulated the secretion of granzyme B and IFN-γ at the tumor site, which thereby significantly improved the antitumor effect.	[137]
Mature DC membrane	Cancer cell membrane coated PLGA nanoparticles	Good biocompatibility and biodegradability, high active targeting efficiency	--	HPV E6- and E7-expressing TC-1, B16-OVA, and Hepa 1–6 cells, multiple mouse models	The biomimetic delivery system could directly cross-prime T cells without the help of APCs and elicit robust antigen-specific T cell immunotherapy.	[138]

**Table 3:** Application of biomimetic cell-derived nanoparticles in cancer vaccines.

Cell Membrane	Core Nanoparticle	Characteristics of the Core Nanoparticle	Drug/Imaging Agent Encapsulated	Cancer Models and the Reason for Selecting Each Cancer Cell Line	Biomedical Application	Ref.
CRT-expressed cancer cell membrane	PLGA nanoparticles	Good biocompatibility and biodegradability, low passive targeting efficiency, rapid clearance by macrophages	Adjuvant R837	Luc-4T1 cells, homologous targeting	The CRT expressed on the surface of the nanovaccine facilitated the internalization of the nanovaccine by DCs. R837 released from the nanovaccine excited TLR7 and further activated DCs.	[145]
Cancer cell membrane	Silicon@Au nanocomposites	Good biocompatibility, low passive targeting efficiency, rapid clearance by macrophages	Au	4T1 cells, homologous targeting	The biomimetic delivery system based on nanocomposites with weak immune stimulation could still induce DCs maturation to activate the downstream anti-tumor immune responses.	[146]
BDVs presenting the neoantigens	--	--	Adjuvant GM-CSF	B16F10-luc cells, leading tumor	The biomimetic delivery system efficiently recruited DCs and promoted the proliferation of TILs and memory T cells.	[150]
Erythrocyte membrane	Manganese-doped imidazolate frameworks-90 nanoparticles	Good biocompatibility, low passive targeting efficiency, rapid clearance by macrophages	DNAzyme	4T1 cells, classic tumor model	The biomimetic delivery system closely mimicked the process of herpesvirus infection, ultimately initiating cGAS-STING pathway-mediated innate immunotherapy.	[154]
Cancer cell membrane	Mesoporous polydopamine nanoparticles	Good biocompatibility and biodegradability, PTT nanoplatform, low passive targeting efficiency, rapid clearance by macrophages	Adjuvant R848	4T1 cells, homologous targeting	The biomimetic delivery system effectively stimulated the immune response and demonstrated excellent photothermal immunotherapy, which significantly promoted the activation and maturation of lymph node DCs, and stimulated CD8+ T cells and memory T cells.	[155]
Bone marrow-derived macrophage (BMDM) membrane	Poly (lactic-co-glycolic acid) nanoparticles (PLP NPs)	Good biocompatibility and biodegradability, low passive targeting efficiency, rapid clearance by macrophages	Adjuvant Poly I: C (PIC)	4T1 cells, classic tumor model	The PIC released from the biomimetic nanoparticles could not only directly induce tumor cell apoptosis, but also polarize exogenous BMDMs into killing M1 macrophages and activate endogenous APCs to trigger robust anti-tumor immune responses.	[156]
Tumor-antigen activated DC membrane	PLGA nanoparticles	Good biocompatibility and biodegradability, low passive targeting efficiency, rapid clearance by macrophages	Rapamycin (RAPA)	C6-LUC cells, biomimetic nanoparticles possessing the potential to cross the BBB	The biomimetic delivery system could activate CD8+ T cells directly or indirectly to reconstitute the glioma tumor immune microenvironment and increase the proportion of NK cells to reduce the exhausted T cells, resulting in a significant anti-glioma efficacy.	[157]
CD80 engineered cancer cell-membrane	--	--	--	B16-OVA cells, homologous targeting	The biomimetic nanoparticle platform could directly activate T cells without the help of professional APCs.	[158]
Anti-CD40 scFv-anchored cancer cell membrane	PLGA nanoparticles	Good biocompatibility and biodegradability, low passive targeting efficiency, rapid clearance by macrophages	--	MC38 and Panc02 cells, homologous targeting, and multiple mouse models	The biomimetic nanoparticle platform effectively promoted DCs maturation in CD40-humanized transgenic mice, improved the engagement and expansion of cognate T-cell immune responses, and facilitated subsequent adaptive immune responses.	[159]
CD47KO/CRT dual-bioengineered cancer cell membrane	Hyperbranched PEI25k nanoparticles	Good biocompatibility, large surface area, low passive targeting efficiency, rapid clearance by macrophages	Unmethylated cytosine-phosphate-guanine (CpG) adjuvant	B16F10 cells, homologous targeting	The biomimetic nanoparticle platform significantly stimulated APCs, resulting in the activation of CD8+ T cells and intense anti-tumor immune responses.	[160]
MHC-I-Ag-anchored mature DC membrane	--	--	aPD-1	B16F10 cells, leading tumor	The biomimetic nanoparticle platform could present neoantigens to CD8+ T cells directly, resulting in strong CTL responses. In addition, the immunosuppressive reversal function of anti-PD-1 antibody was enhanced by CD28/B7 co-stimulation.	[161]
Cancer cell membrane	Poloxamer 407 nanoparticles	Good biocompatibility, low passive targeting efficiency, rapid clearance by macrophages	Adjuvant R837	HCT116 cells, homologous targeting	The biomimetic nanoparticle platform was presented with APCs to secret immunofactors and to activate the lymphatic immune network, resulting in significant tumor regression.	[162]
Cancer cell membrane	Manganese dioxide (MnO_2_) nanoparticles	Good biocompatibility, low passive targeting efficiency, rapid clearance by macrophages	Polythiophene	B16F10 cells, homologous targeting	The biomimetic nanoparticle platform could stimulate a specific T cell anti-tumor immune response by promoting APCs maturation, autologous tumor antigen presentation, as well as generating local microinflammation.	[163]
Cancer cell membrane expressing fibroblast activation protein-α (FAP)	--	--	--	CT26, B16F10, LLC, and 4T1 cells, 90% of human tumor tissues overexpressing FAP and multiple mouse models	The biomimetic nanoparticle platform suppressed tumor growth by triggering robust and specific T cell anti-tumor immune responses. Moreover, the biomimetic nanoparticle platform facilitated tumor ferroptosis by releasing IFN-γ from CTLs and eliminating FAP+CAFs.	[164]

**Table 4:** Application of biomimetic cell-derived nanoparticles in modulating the ITME.

Cell Membrane	Core Nanoparticle	Characteristics of the Core Nanoparticle	Drug/Imaging Agent Encapsulated	Cancer Models and the Reason for Selecting Each Cancer Cell Line	Biomedical Application	Ref.
Bacterial outer membrane	PLGA nanoparticles	Good biocompatibility and biodegradability, low passive targeting efficiency, rapid clearance by macrophages	DOX and R848	4T1 cells, classic tumor model	The biomimetic nanoparticle platform could polarize M2 macrophages into M1 macrophages, promote the secretion of TNF-α and IL-6, which thereby activated the anti-tumor immune responses.	[176]
Cancer cell membrane overexpressing SIRPα variants	Magnetic nanoparticles	Good biocompatibility, magnetic targeting, rapid clearance by macrophages	--	B16F10 and 4T1 cells, homologous targeting, and multiple mouse models	In this system, the gCM shell genetically overexpressing SIRPα variants efficiently blocked the CD47-SIRPα pathway, while the nanoparticle cores polarized M2 macrophages into M1 macrophages, which thereby synergistically facilitated macrophage phagocytosis of tumor cells and triggered T-cell immune response.	[177]
Erythrocyte membrane	Amphiphilic PCL nanoparticles	Good biocompatibility and biodegradability, low passive targeting efficiency, rapid clearance by macrophages	DOX	4T1 cells, classic tumor model	The Hb moiety of the biomimetic nanoparticle platform could effectively kill the tumor cells. In addition, the O_2_ released by Hb alleviated cancer hypoxia and further augmented the anti-tumor immune response.	[181]
4T1 and 3T3 hybrid cell membrane	Solid lipid nanoparticles	Good biocompatibility and biodegradability, low passive targeting efficiency, rapid clearance by macrophages	PTX and glycolysis inhibitor PFK15	4T1 cells, homologous targeting	The biomimetic nanoparticle platform possessed outstanding dual-targeting ability. The encapsulated PFK15 could inhibit glycolysis in both CAFs and cancer cells, resulting in elevated immune responses.	[185]
Cancer cell membrane	PLGA nanoparticles	Good biocompatibility and biodegradability, low passive targeting efficiency, rapid clearance by macrophages	ICG and DCT	4T1 cells, homologous targeting	ICG induced a sharp increase in cytosolic Ca^2+^ concentration, promoted cytochrome *c* release and subsequent caspase-3 activation. DCT synergistically up-regulated the expression of gasdermin E by inhibiting DNA methylation, enhancing the cleavage of gasdermin E by caspase-3, and causing robust cancer pyroptosis.	[188]
Macrophage cell-derived sEVs	--	--	siRNA against YTHDF1	MGC-803 and HGC-27 cells, YTHDF1 promoting tumor progression in a variety of cancers and multiple mouse models	This nanosystem efficiently depleted YTHDF1 expression and stimulated strong cytotoxic T lymphocytes responses through hampering frizzled7 translation and inactivating the Wnt/β-catenin pathway in an m6A dependent manner.	[189]
Platelet membrane	PLA nanoparticles	Good biocompatibility and biodegradability, low passive targeting efficiency, rapid clearance by macrophages	R848	MC38 and 4T1 cells, multiple mouse models	The biomimetic nanoparticle platform promoted the robust activation of APCs and increased immune infiltration, resulting in complete tumor regression.	[190]
Activated neutrophils membrane	Redox-responsive polymer nanoparticles	Good biocompatibility and biodegradability, low passive targeting efficiency, rapid clearance by macrophages	DOX	4T1 cells, classic tumor model	The biomimetic nanoparticle platform could prevent the recruitment and functions of MDSCs, thereby inhibiting tumor recurrence and metastasis.	[191]
Tumor-associated macrophage membrane with high macrophage colony-stimulating factor 1 receptor (CSF1R)	Rare-earth-upconversion nanoparticles	Good biocompatibility, “nanotransducer”, theranostic nanoplatform, low passive targeting efficiency, rapid clearance by macrophages	Rose Bengal	4T1 cells, classic tumor model	The biomimetic nanoparticle platform could block the interaction between TAMs and cancer cells via depleting the CSF1 secreted by cancer cells. In addition, the biomimetic nanoparticle platform-mediated photodynamic therapy (PDT) could polarize M2 macrophages into M1 macrophages.	[192]
Erythrocyte membrane	Copper peroxide (CP) nanoparticles	Good biocompatibility, PDT nanoplatform, low passive targeting efficiency, rapid clearance by macrophages	H_2_O_2_ and protoporphyrin (PpIX)	B16F10 cells, leading tumor	The biomimetic nanoparticle platform could relieve tumor hypoxia and polarize M2 macrophages into M1 macrophages. Furthermore, the ROS generated by photosensitizers could cause the ICD of tumor cells.	[193]
Macrophage cell membrane	Polydopamine nanoparticles	Good biocompatibility and biodegradability, PTT nanoplatform, low passive targeting efficiency, rapid clearance by macrophages	TMP195 (a TAMs repolarization agent)	4T1 cells, classic tumor model	The loaded TMP195 significantly polarized M2 macrophages into M1 macrophages and subsequently remodeled the ITME in residual tumor after PTT, which thereby rescued CTLs.	[194]
Cancer cell membrane	Cu_2_-xSe nanoparticles	Good biocompatibility, effective ferroptosis, low passive targeting efficiency, rapid clearance by macrophages	Indoximod (IND) and JQ1	GL261 cells, homologous targeting	The smart nanoparticle platform could remodel the ITME by increasing M1 macrophages and preventing Tregs cell infiltration. In addition, it also served as a checkpoint inhibitor to inhibit the expression of PD-L1 on cancer cells.	[195]
M1-like macrophage cell membrane	--	--	Celastrol	LLC and GL261 cells, multiple mouse models	M1 macrophages not only acted as drug delivery carriers for celastrol but also as therapeutic agents. In turn, celastrol could maintain the anticancer polarization of M1 macrophages.	[196]
Erythrocyte membrane	TiO_2_ nanoparticles	Good biocompatibility, SDT nanoplatform, low passive targeting efficiency, rapid clearance by macrophages	Hb and RRx-001	4T1 cells, classic tumor model	Upon reaching the hypoxic TME, the Hb in this smart nanoparticle platform was deoxygenated, further initiating a series of reactions that generated a large number of RNS in cascade fashion. Subsequently, oxygen compensation by Hb enhanced TiO_2_-mediated ROS for SDT, and the resulting highly active RNS triggered ICD in cancer cells.	[197]
M1-like macrophage cell membrane	Magnetic polymer nanoparticles	Good biocompatibility and biodegradability, magnetic targeting, rapid clearance by macrophages	R837	4T1 cells, classic tumor model	The intracellular uptake of this smart nanoparticle platform could greatly polarize M2 macrophages into M1 macrophages with the synergy of R837 and Fe_3_O_4_, alleviating the immunosuppression of the TME for immune recovery.	[198]
Cancer cell membrane	Fe-doped polydiaminopyridine nanoparticles	Good biocompatibility, SDT nanoplatform, low passive targeting efficiency, rapid clearance by macrophages	Ce6	4T1 cells, homologous targeting	The prepared smart nanoparticle platform could significantly reduce the unnecessary consumption of H_2_O_2_ in the TME, while US irradiation could promote the exposure of Fe-PDAP and provide more adequate O_2_ supply, enabling efficient ROS production and DCs maturation.	[199]

**Table 5:** Application of biomimetic cell-derived nanoparticles in combination therapy.

Cell Membrane	Core Nanoparticle	Characteristics of the Core Nanoparticle	Drug/Imaging Agent Encapsulated	Cancer Models and the Reason for Selecting Each Cancer Cell Line	Biomedical Application	Ref.
Anti-PD-L1 scFv-anchored HEK 293T cell membrane	Polydopamine-modified gold nanoparticles	Good biocompatibility and biodegradability, PTT nanoplatform, low passive targeting efficiency, rapid clearance by macrophages	Au and membrane-bound aPD-L1 scFv	MC38 cells, immunotherapy for colorectal cancer	The developed smart nanoparticle platform combined PD-1/PD-L1 blockade with PTT, which triggered the immune stimulatory responses and remodeled the ITME.	[202]
Anti-DEC205 anchored cancer cell membrane	Porphyrin-based metal organic frameworks (MOFs)	Good biocompatibility and biodegradability, SDT nanoplatform, low passive targeting efficiency, rapid clearance by macrophages	R848 and AuPt	Hep1-6 cells, homologous targeting	AMR-MOF@AuPt-mediated multimodal therapies based on sonoimmunotherapy induced systemic immune responses and long-term memory immunity.	[203]
Cancer cell membrane anchored with a cleavable peptide by MMP2	PLGA nanoparticles	Good biocompatibility and biodegradability, low passive targeting efficiency, rapid clearance by macrophages	Afatinib (AFT) and 2-bromopalmitate (2-BP)	4T1 cells, homologous targeting	The developed smart nanoparticle platform combined targeted therapy with immunotherapy, which remarkably prevented tumor growth and metastasis.	[204]
Hybrid pH-sensitive membrane	Mesoporous silica nanoparticles	Good biocompatibility and biodegradability, large surface area, low passive targeting efficiency, rapid clearance by macrophages	R837 and Au	4T1 cells, homologous targeting	The developed smart nanoparticle platform realized the synergistic effect of starvation/PTT/immunotherapy and induced a long-term immune memory effect.	[205]
Macrophage cell membrane	Bacterial outer membrane vesicles	Good biocompatibility and biodegradability, potent anti-cancer potential, low targeting efficiency	Ce6 and DOX	4T1 cells, classic tumor model	The developed smart nanoparticle platform realized the synergism of PDT/chemo-/immunotherapy.	[206]
Hybrid cell membrane	Glutathione (GSH) decorated Te nanoparticles	Good biocompatibility, radiotherapy sensitization, radiotherapy nanoplatform, low passive targeting efficiency, rapid clearance by macrophages	GTe	4T1 cells, homologous targeting	The developed smart nanoparticle platform realized the synergism of radiotherapy and immunotherapy.	[207]
DC-derived sEVs	MBPN-TCyP nanoparticle complexes	Good biocompatibility, PDT nanoplatform, mitochondrion-targeting, rapid clearance by macrophages	MBPN-TCyP (an AIE photosensitizer)	4T1 and CT26 cells, multiple mouse models	The developed smart nanoparticle platform realized the synergism of PDT/immunotherapy and inhibited the self-renewal of cancer stem cells for tumor suppression.	[208]
Cancer cell membrane	Hollow manganese dioxide (HMnO_2_) nanoparticles	Good biocompatibility, GSH-sensitive, excellent loading capacity, low passive targeting efficiency, rapid clearance by macrophages	Ginsenoside Rh2 (Rh2)	K7M2 cells, homologous targeting	The developed smart nanoparticle platform realized attractive immuno-chemo-dynamic combined therapeutic efficiency.	[209]
Stem cell membrane	Polydopamine nanoparticles	Good biocompatibility and biodegradability, PTT nanoplatform, low passive targeting efficiency, rapid clearance by macrophages	DOX and PD-L1 siRNA	PC-3 cells, one of the major tumors	The developed smart nanoparticle platform showed attractive immuno-chemo-dynamic combined therapeutic efficiency for PCa bone metastases.	[210]
M1-like macrophage cell membrane	Polyethylenimine (PEI) nanoparticles	Good biocompatibility, large surface area, rapid clearance by macrophages	DOX and short-hairpin RNA (shRNA) targeting Ptpn2	B16F10 cells, leading tumor	The developed smart nanoparticle platform realized the synergism of chemotherapy and gene immunotherapies.	[211]
M1-like macrophage cell membrane	Magnetic photothermal nanocomplexes	Good biocompatibility, PTT nanoplatform, low passive targeting efficiency, rapid clearance by macrophages	DOX	4T1 cells, classic tumor model	The developed smart nanoparticle platform enhanced ROS generation and exhibited outstanding chemo-phototherapy efficacy.	[212]
Cancer cell membrane	Mesoporous polydopamine nanoparticles	Good biocompatibility and biodegradability, PTT nanoplatform, low passive targeting efficiency, rapid clearance by macrophages	Chloroquine (CQ, an autophagy inhibitor)	RM-1 cells, homologous targeting	The developed smart nanoparticle platform facilitated PTT and autophagy blockade for synergistic tumor elimination.	[213]
Hybrid cell membrane	Polydopamine nanoparticles	Good biocompatibility and biodegradability, PTT nanoplatform, low passive targeting efficiency, rapid clearance by macrophages	--	B16F10 cells, homologous targeting	The developed smart nanoparticle platform realized the synergism of OMV-mediated immunotherapy and hollow polydopamine-mediated PTT.	[84]
Anti-PD-1 peptide AUNP-12 modified cancer cell membrane	Dendritic large-pore mesoporous silica nanoparticles	Good biocompatibility and biodegradability, low passive targeting efficiency, rapid clearance by macrophages	Copper sulfide (CuS) and R848	4T1 cells, homologous targeting	The developed smart nanoparticle platform realized the synergism of PDT and immunotherapy.	[214]
Cancer cell membrane expressing CD86 and anti-LAG3	Polymeric nanoparticles	Good biocompatibility, PDT nanoplatform, mitochondrion-targeting, rapid clearance by macrophages	Fs (an AIE photosensitizer)	4T1 cells, homologous targeting	The developed smart nanoparticle platform realized the synergism of PDT and immunotherapy by directly presenting tumor antigens and reversed immunosuppression.	[215]
Cancer cell membrane	Liposomes	Good biocompatibility and biodegradability, pH-sensitive, low passive targeting efficiency, rapid clearance by macrophages	RA-V (a chemotherapeutic drug) and BMS202	CT26 cells, homologous targeting	The developed smart nanoparticle platform systemically eliminated hypoxia tumors by synergistic chemotherapy and checkpoint blockade immunotherapy.	[216]
Cancer cell membrane	Ovalbumin antigen (OVA) nanoparticles	Good biocompatibility and biodegradability, rapid clearance by macrophages	Ce6	B16-OVA cells, homologous targeting	The developed smart nanoparticle platform facilitated ROS-triggered immune cascade for photodynamic immunotherapy.	[217]
M1-like macrophage cell membrane	Nanoparticle complexes	Good biocompatibility, laser-sensitive, size-changeable, rapid clearance by macrophages	Ce6, DOX, and d indoleamine 2,3-dioxygenase 1 inhibitor	4T1 and B16F10 cells, multiple mouse models	The developed smart nanoparticle platform realized the synergism of PDT/chemo-/immunotherapy.	[218]
Erythrocyte membrane	PLGA nanoparticles	Good biocompatibility and biodegradability, low passive targeting efficiency, rapid clearance by macrophages	P2-PPh3 (an AIE photosensitizer) and Poly (I:C)	B16F10 cells, leading tumor	The developed smart nanoparticle platform combined the PDT properties of P2-PPh3 with the immunotherapy properties of Poly (I:C).	[219]

## Data Availability

No new data were created or analyzed in this study. Data sharing is not applicable for this article.
